# A prediction model for worsening diabetic retinopathy after panretinal photocoagulation

**DOI:** 10.1186/s13098-022-00892-z

**Published:** 2022-08-26

**Authors:** Jinglan Li, Xuanlong Li, Mingxing Lei, Wanyue Li, Wenqian Chen, Tianju Ma, Yi Gao, Zi Ye, Zhaohui Li

**Affiliations:** 1 Medical School of Chinese PLA, Beijing, China; 2 Department of Ophthalmology, The First Medical Centre, Chinese PLA General Hospital, 28 Fuxing Road, Haidian District, Beijing, China

**Keywords:** Diabetic retinopathy, Panretinal photocoagulation, Risk factors, Prediction model

## Abstract

**Background:**

As one of the severe complications of diabetes mellitus, diabetic retinopathy (DR) is the leading cause of blindness in the working age worldwide. Although panretinal photocoagulation (PRP) was standard treatment, PRP-treated DR still has a high risk of progression. Hence, this study aimed to assess the risk factors and establish a model for predicting worsening diabetic retinopathy (DR-worsening) within five years after PRP.

**Methods:**

Patients who were diagnosed with severe non-proliferative diabetic retinopathy or proliferative diabetic retinopathy and treated with PRP were included, and those patients were randomly assigned to either a training or validation cohort. The multivariate logistic regression analysis was used to screen potential risk factors for DR-worsening in the training cohort. Then the model was established after including significant independent risk factors and further validated using discrimination and calibration.

**Results:**

A total of 271 patients were included, and 56.46% of patients had an outcome of DR-worsening. In the training cohort (n = 135), age (odds ratio [OR] = 0.94, 95% confidence interval [CI] 0.90–0.98), baseline best corrected visual acuity (logMAR) (OR = 10.74, 95% CI 1.84–62.52), diabetic nephropathy (OR = 9.32, 95% CI 1.49–58.46), and hyperlipidemia (OR = 3.34, 95% CI 1.05–10.66) were screened out as the independent risk factors, which were incorporated into the predictive model. The area under the receiver operating characteristic curve and calibration slope in the training and validation cohort were 0.79, 0.96 (95% CI 0.60–1.31), and 0.79, 1.00 (95% CI 0.66–1.34), respectively. Two risk groups were developed depending on the best cut-off value of the predicted probability, and the actual probability was 34.90% and 82.79% in the low-risk and high-risk groups, respectively (*P* < 0.001).

**Conclusions:**

This study developed and internally validated a new model to predict the probability of DR-worsening after PRP treatment within five years. The model can be used as a rapid risk assessment system for clinical prediction of DR-worsening and identify individuals at a high risk of DR-worsening at an early stage and prescribe additional treatment.

**Supplementary Information:**

The online version contains supplementary material available at 10.1186/s13098-022-00892-z.

## Background

Diabetic retinopathy (DR), one of the most common microvascular complications of diabetes mellitus, is the leading cause of blindness and visual impairment in the working age (20–65 years) worldwide [1, 2]. It is classified as non-proliferative diabetic retinopathy (NPDR) or proliferative diabetic retinopathy (PDR) based on the proliferative status of retinal neovascularization [3]. PDR could be followed by serious complications, such as vitreous hemorrhage, tractional retinal detachment, and neovascular glaucoma, a more advanced stage with a risk of poor vision outcome [4, 5].

Panretinal photocoagulation (PRP) is currently the standard treatment for PDR and severe NPDR, which was recommended in previous clinical trials [6, 7]. With proper treatment including PRP, PDR patients have a 90% reduced risk of blindness within five years [8]. However, PRP is far from a “one-and-done treatment”, 45% of the eyes required supplemental PRP, intravitreal anti-vascular endothelial growth factor (VEGF) injection, or even vitrectomy, within two years after PRP treatment [9, 10]. Those patients usually suffered from very poor visual prognosis, even blindness [11–13]. Identification of risk factors associated with progression after PRP would be beneficial to guide preventive and therapeutic strategies among PRP-treated DR patients.

Currently, several risk factors have been proposed to be associated with DR-worsening, including age, uncontrolled diabetes, renal dysfunction, lipid metabolic abnormalities, anemia, etc. [14–18]. Although these factors can provide guidance in clinical management, they cannot accurately predict the specific risk of DR-worsening. In addition, studies on prediction of the prognosis of PRP are very scarce, and without a definitive conclusion. Risk factors identified from DR-worsening patients might not be applicable among PRP-treated DR patients. Thus, it is of great clinical significance to investigate the potential risk factors associated with DR-worsening in particular after PRP treatment. Furthermore, to achieve risk stratification and subsequently perform individualized preventions, it is urgent to develop a model to predict the risk probability of DR-worsening specifically among this population.

Therefore, this study aimed to develop a model to predict the progression of DR after PRP to prevent it better and earlier. This study speculated that the model could reflect the relationships between DR-worsening and its potential risk factors and quantify the contribution of these factors by correlation coefficients.

## Methods

### Study population

**Fig. 1:**
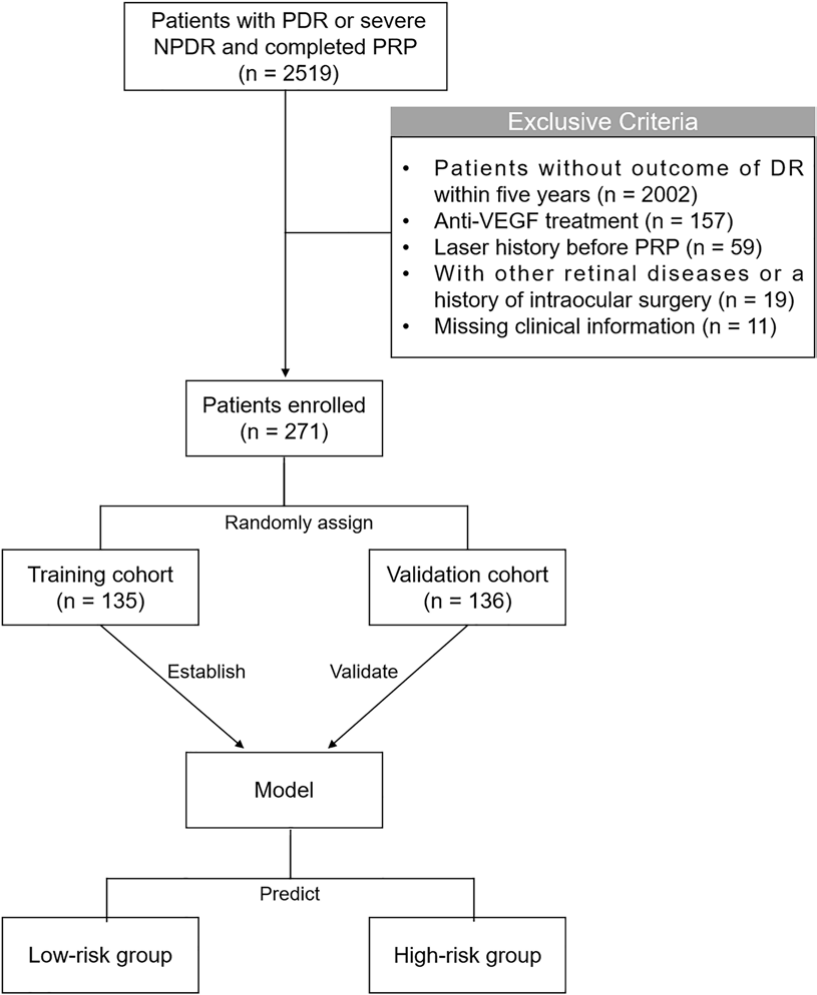
The flow chart

The patients included in our study were all diagnosed with PDR or severe NPDR and treated with PRP at Chinese PLA General Hospital between 1 January 2008 and 1 January 2021 (n = 2519). Patient's clinical data were extracted and collected from the hospital electronic medical record system. Only one eye of every patient was included in the study, and the eye with more severe DR or lower vision was included if both two eyes met the criteria for inclusion. The data were collected and recorded by two experienced ophthalmologists to guarantee data quality. When disagreements occurred, they were resolved through discussion. Patients were excluded if they met any of the following criteria: (1) Missing the outcome of DR within five years; (2) Received anti-VEGF treatment before or after PRP; (3) Having a history of the laser before PRP; (4) Having the history of intraocular surgery other than cataract surgery; (5) Having the history of other retinal diseases, such as age-related macular degeneration, retinal artery/vein occlusion, ischemic optic neuropathy, posterior uveitis, glaucoma, or other eye diseases that affected fundus examination; (6) Missing clinical information. After exclusion, 271 patients were included in this study (Fig. 1).

Treatment of severe NPDR and PDR was performed according to guidelines [19]. According to ETDRS protocol [20], a standard argon-type laser was used in PRP, with the recommended settings including 1200 to1600 spots, approximately 400 μm burning in size, 200 mW power, and 100 ms pulse duration. PRP was administered across four treatment sessions, one session per week. Finally, laser burn spots were scattered evenly across the retina almost to the equator and away from the macula [20].

This study was conducted in accordance with the principles of the Declaration of Helsinki, and the study protocol was approved by the Ethics Committee of Chinese PLA General Hospital (no. S2021-068-01). Patient consent for inclusion was waived because all data were anonymized and the study was retrospective in nature.

### Outcomes and definitions

The positive outcome was DR-worsening within five years after PRP treatment. The patient was considered to have a positive outcome if any of the following situations occurred to them within five years: vitreous hemorrhage, tractional retinal detachment, neovascular glaucoma, requiring further PRP, intravitreal anti-VEGF injection, or vitrectomy. The negative outcome was non-DR-worsening, meaning that the above conditions did not occur within five years, and the vision remained stable (the decline was not more than two lines). Patients were followed until the occurrence of the outcome, loss to follow-up, or administrative censoring, whichever came first. The last follow-up date was 1 August 2021.

### Risk factors

The study included the following 29 potential risk factors for predicting post-PRP progression of DR: (1) Ocular parameters: stage of diabetic retinopathy (PDR or severe NPDR); baseline best corrected visual acuity (BCVA); (2) Clinical case data: age; sex; type of diabetes (type 1 or type 2); diabetes duration; diabetic nephropathy; diabetes neuropathy; coronary heart disease; prior stroke; hyperlipidemia; grade of hypertension (0–3); body mass index; (3) Laboratory parameters: homocysteine; fasting blood glucose; urea; creatinine; uric acid; total cholesterol; triglyceride; high-density lipoprotein; low-density lipoprotein; serum superoxide dismutase; glycosylated serum protein; serum cystatin C; hemoglobin; hematocrit; platelet; neutrophil/lymphocyte ratio.

Ocular parameters were assessed at baseline by recording BCVA, intraocular pressure, slit-lamp examination, retinal examination, and fundus photograph. Baseline BCVA was assessed with the Snellen chart and expressed in logMAR values. Detailed fundus examination was performed by the trained ophthalmologist using direct and indirect ophthalmoscope. Fundus fluorescein angiography was performed before laser treatment to identify suspicious but clinically insignificant retinal neovascularization which was the most reliable and important evidence for the PDR. Macular OCT can determine macular edema or other macular lesions, and B ultrasound can determine retinal detachment and fibrous membrane hyperplasia. Ophthalmic evaluation was conducted by a single retina specialist, stereoscopic fundus photography and fundus fluorescein angiography were conducted by a single examiner.

Diabetic nephropathy was defined as urinary albumin creatinine ratio ≥ 30 mg/g in the absence of other primary causes of kidney damage [21]. Diabetic neuropathy was tested with a 128-Hz tuning fork for vibration sense and a 10-g monofilament test for light touch perception (on four sites per foot) [22]. Hyperlipidemia was defined as total cholesterol ≥ 6.2 mmol/L or triglyceride ≥ 2.3 mmol/L or low-density lipoprotein ≥ 4.1 mmol/L or high-density lipoprotein < 1.0 mmol/L [23]. Coronary heart disease and prior stroke were judged by inquiring about the medical history and referring to their medical records. Venous blood was taken from all patients on an empty stomach to detect biochemistry and blood routine.

### Statistical analysis

In the study, normally distributed continuous variables were expressed as mean ± standard deviation, non-normally distributed continuous variables were presented as median with the quartile range, and categorical variables were summarized as proportion (%). Using the Kolmogorov–Smirnov test or Shapiro–Wilk test to determine if each variable had a normal distribution. Baseline characteristics of patients were compared between groups using the Chi-square test for categorical variables, the Student’s T-test or the Mann–Whitney test for continuous variables, as appropriate. Statistical analyses were performed using SAS software version 9.4 (SAS Institute Inc., Cary, NC) and IBM SPSS 23.0 (IBM Corp, Armonk, NY, USA) for Windows XP. *P* < 0.05 was considered statistically significant (* *P* < 0.05, ** *P* < 0.01).

All the included patients were randomly assigned to either the training cohort or validation cohort. Univariate and multivariate logistic regression analysis was respectively used to analyze the potential risk factors of DR-worsening in the training cohort, and the risk factors identified by the univariate analysis (*P* < 0.20) entered into the multivariate analysis. The model was established depending on the training cohort subsequently, and significant risk factors identified by the multivariate analysis (stepwise selection) were enrolled to create the model. The model was developed as follows:

$$P (Y=1)=e^{(intercept+ax1+bx2+cx3+dx4)}/(1+e^{(intercept+ax1+bx2+cx3+dx4)} ).$$

*P* (*Y* = 1) represented the predicted probability of DR-worsening, and $a$, $b$, $c$, and $d$ were the corresponding estimates of the included factors.

Discrimination and calibration in both training and validation cohorts were used to evaluate the performance of the model. The discriminative capability of the model to distinguish patients with and without DR-worsening was mainly assessed by the area under the receiver operating characteristic curve (AUROC) and discrimination slope. The discriminative slope was defined as the mean difference in the predicted probabilities of developing DR-worsening between patients actually with DR-worsening and without DR-worsening. Besides, the correct classification rate (CCR), sensitivity, and specificity were also used to evaluate the formula’s discrimination. Furthermore, the consistency between predicted and actual observed probability of DR-worsening was defined as the calibration of the model, and it was evaluated based on the calibration curve. In addition, the formula’s calibration was also evaluated by the Hosmer–Lemeshow goodness-of-fit test, and *P* > 0.05 in the goodness-of-fit statistics indicated a favorable match between predicted and observed actual probability of DR-worsening.

Each patient had an actual probability of DR-worsening and a predicted probability that was calculated using the model. Based on the best cut-off value of the predicted probability depending on the largest sum of sensitivity and specificity, all patients were divided into a low-risk group and a high-risk group. We further calculated and compared the difference between actual probability of DR-worsening of the two risk groups.

## Results

### Patient’s characteristics

A total of 271 patients were enrolled with a mean age of 50.69 ± 11.24 years and 58.30% of them were men. The majority of comorbidity was hypertension (73.80%), followed by hyperlipidemia (27.68%), diabetic nephropathy (26.57%), coronary heart disease (8.86%), diabetic neuropathy (8.12%), and prior stroke (5.17%). The demographics and laboratory parameters of patients are presented in Table 1. The incidence of DR-worsening was 56.46%, and 153 patients were included in the DR-worsening group, including 112 eyes with vitreous hemorrhage, 26 eyes with vitreous hemorrhage and retinal detachment, seven eyes with secondary glaucoma and retinal detachment, four eyes with vitreous hemorrhage and macular edema, two eyes with secondary glaucoma and macular edema, and two eyes with secondary glaucoma, within five years after PRP treatment.

**Table 1:** Patients’ baseline characteristics

Variables	Total patients (n = 271)	DR-worsening (n = 153)	Non-DR-worsening (n = 118)	*P* value^a^
Age, years	50.69 ± 11.24	49.16 ± 10.90	52.66 ± 11.42	0.011*
Male, n (%)	158 (58.30%)	95 (62.1%)	63 (53.4%)	0.150
Baseline BCVA (logMAR)	0.30 (0.10, 0.52)	0.40 (0.22, 0.70)	0.22 (0.10, 0.40)	< 0.001**
Diabetes duration, years	12.24 ± 7.37	11.92 ± 7.26	12.64 ± 7.52	0.524
Type 2 diabetes, n (%)	261 (96.31%)	145 (94.8%)	116 (98.3%)	0.127
PDR, n (%)	169 (62.36%)	98 (64.1%)	71 (60.2%)	0.324
Diabetic nephropathy, n (%)	72 (26.57%)	65 (42.5%)	7 (5.9%)	< 0.001**
Diabetic neuropathy, n (%)	22 (8.12%)	19 (12.4%)	3 (2.5%)	0.003**
Coronary heart disease, n (%)	24 (8.86%)	17 (11.1%)	9 (7.6%)	0.335
Prior Stroke, n (%)	14 (5.17%)	8 (5.2%)	6 (5.1%)	0.958
Hyperlipidemia, n (%)	75 (27.68%)	59 (38.6%)	16 (13.6%)	< 0.001**
*Hypertension, n (%)*				
0	71 (26.20%)	40 (26.1%)	31 (26.3%)	0.111
1	83 (30.63%)	41 (26.8%)	42 (35.6%)	
2	48 (17.71%)	23 (15.0%)	25 (21.2%)	
3	69 (25.46%)	49 (32.0%)	20 (16.9%)	
BMI, kg/m^2^	25.52 ± 3.42	25.46 ± 3.67	25.60 ± 3.09	0.732
Homocysteine, umol/L	13.34 ± 7.91	14.14 ± 9.40	12.29 ± 5.27	0.061
Fasting blood glucose, mmol/L	6.96 ± 2.77	7.18 ± 2.81	6.67 ± 2.71	0.085
Urea, umol/L	6.66 ± 3.86	7.58 ± 4.78	5.47 ± 1.47	< 0.001**
Creatinine, mmol/L	71.40 (56.70, 92.50)	76.20 (61.15, 110.50)	66.30 (54.83, 79.75)	< 0.001**
Uric acid, umol/L	327.98 ± 90.23	340.59 ± 89.91	311.64 ± 88.37	0.009**
Total cholesterol, mmol/L	4.36 ± 1.18	4.49 ± 1.36	4.18 ± 0.89	0.090
Triglyceride, mmol/L	1.18 (0.88, 1.75)	1.27 (0.87, 1.98)	1.15 (0.89, 1.57)	0.139
High-density lipoprotein, mmol/L	1.13 ± 0.34	1.13 ± 0.35	1.12 ± 0.32	0.92
Low-density lipoprotein, mmol/L	2.76 ± 0.97	2.82 ± 1.10	2.67 ± 0.78	0.281
Serum superoxide dismutase, U/ML	146.23 ± 27.01	144.44 ± 30.87	148.56 ± 20.87	0.076
Glycosylated serum protein, umol/L	228.91 ± 75.04	228.53 ± 82.28	229.39 ± 64.80	0.986
Serum cystatin C, mg/L	1.04 (0.87, 1.27)	1.11 (0.89, 1.41)	0.97 (0.86, 1.11)	< 0.001**
Hemoglobin, g/L	129.58 ± 18.91	127.04 ± 20.54	132.88 ± 16.07	0.044*
Hematocrit	0.38 ± 0.05	0.37 ± 0.06	0.39 ± 0.04	0.026*
Platelet, 10^9^/L	215.19 ± 61.32	213.56 ± 64.11	217.31 ± 57.69	0.613
Neutrophil/lymphocyte ratio	1.91 (1.47, 2.57)	1.97 (1.53, 2.76)	1.81 (1.39, 2.25)	0.017*

Values are expressed as mean ± SD, median with inter-quartile range, or n (%)

*BCVA* best corrected visual acuity, *DR* diabetic retinopathy, *PDR* proliferative diabetic retinopathy, *BMI* body mass index

*P < 0.05, **P < 0.01

^a^ For comparison between DR-worsening and non-DR-worsening

Compared with the non-DR-worsening group, patients with the DR-worsening outcome were more likely to be younger, have lower baseline BCVA (or higher logMAR BCVA), have more frequency of diabetic nephropathy, diabetic neuropathy, and hyperlipidemia, and the differences in laboratory parameters related to renal function (creatinine, urea, uric acid, serum cystatin C) and anemia (hemoglobin, hematocrit) were also statistically significant (*P* < 0.05). In addition, the proportion of males and PDR, fasting blood glucose, homocysteine, and neutrophil/lymphocyte ratio was higher in patients with DR-worsening, and regarding blood lipids, total cholesterol, triglycerides, and low-density lipoprotein were also higher in patients with DR-worsening *vs*. non-DR-worsening, but the differences were not statistically significant (*P* > 0.05). The detail about the comparison of DR-worsening and non-DR-worsening is presented in Table 1.

### Model development

The baseline characteristics of patients in the training cohort (n = 135) and validation cohort (n = 136) are shown in Additional file 1: Table S1. There were no statistically significant differences between the training cohort and the validation cohort in all the 29 risk factors (*P* > 0.05), indicating that patients in the two cohorts were comparable. In the training cohort, univariate analysis was used to analyze the potential risk factors of DR-worsening, then multivariate analysis was conducted after including 15 variables (*P* < 0.20 according to the univariate analysis), and the significant risk factors were defined by the result of multivariate analysis finally. The results of univariate and multivariate logistic regression analysis are shown in Table 2. It indicated that the following factors were all independent risk factors for DR-worsening: age (OR = 0.94, 95% CI 0.90–0.98), baseline BCVA (logMAR) (OR = 10.74, 95% CI 1.84–62.52), diabetic nephropathy (OR = 9.32, 95% CI 1.49–58.46), and hyperlipidemia (OR = 3.34, 95% CI 1.05–10.66). All the above four variables were considered and incorporated into the construction of the predictive model of DR-worsening depending on the result of stepwise regression. Finally, a model was developed as presented in Table 3. According to the model, lower age, lower baseline BCVA (or higher logMAR BCVA), diabetic nephropathy, and hyperlipidemia were associated with a significantly higher incidence of DR-worsening, which was in line with the result of COX regression analysis (Additional file 1: Table S2).

**Table 2:** Univariate and multivariate analysis of patient’s characteristics for predicting DR-worsening

Variables	Univariate analysis		Multivariate analysis	
	OR 95% CI	*P* value	OR 95% CI	*P* value
Age	0.95 (0.93, 0.98)	0.003**	0.94 (0.90, 0.98)	0.006**
Sex	0.62 (0.31, 1.24)	0.177	0.72 (0.25, 2.07)	0.550
Baseline BCVA (logMAR)	6.72 (2.78, 16.25)	< 0.001**	10.74 (1.84, 62.52)	0.008**
Diabetes duration	0.99 (0.94, 1.04)	0.679		
Type of diabetes	0.23 (0.03, 2.02)	0.185	0.59 (0.03, 10.07)	0.712
Stage of DR	1.64 (0.80, 3.37)	0.180	0.96 (0.36, 2.51)	0.928
Diabetic nephropathy	11.11 (3.17, 38.89)	< 0.001**	9.32 (1.49, 58.46)	0.017*
Diabetic neuropathy	4.09 (0.85, 19.68)	0.079	2.48 (0.33, 18.64)	0.377
Coronary heart disease	1.36 (0.42, 4.39)	0.609		
Prior Stroke	0.82 (0.11, 5.99)	0.845		
Hyperlipidemia	4.40 (1.89, 10.24)	0.001**	3.34 (1.05, 10.66)	0.042*
Hypertension	1.11 (0.82, 1.50)	0.512		
BMI	0.99 (0.90, 1.08)	0.810		
Homocysteine	1.02 (0.97, 1.07)	0.537		
Fasting blood glucose	1.07 (0.94, 1.22)	0.318		
Urea	1.01 (1.00, 1.02)	0.049*	1.21 (0.89, 1.64)	0.231
Creatinine	1.41 (1.14, 1.74)	0.002**	1.00 (0.99, 1.01)	0.883
Uric acid	1.00 (1.00, 1.01)	0.053	1.00 (0.99, 1.00)	0.251
Total cholesterol	1.22 (0.90, 1.65)	0.198	0.83 (0.52, 1.31)	0.928
Triglyceride	1.18 (0.87, 1.61)	0.287		
High-density lipoprotein	1.24 (0.48, 3.24)	0.657		
Low-density lipoprotein	1.10 (0.77, 1.58)	0.591		
Serum superoxide dismutase	1.00 (0.99, 1.02)	0.879		
Glycosylated serum protein	1.00 (1.00, 1.01)	0.347		
Serum cystatin C	3.31 (1.18, 9.31)	0.023*	0.66 (0.12, 3.63)	0.636
Hemoglobin	0.99 (0.97, 1.01)	0.148	0.95 (0.85, 1.07)	0.392
Hematocrit	0.00 (0.00, 2.67)	0.095	> 10.00 (0.001- > 10.00)	0.254
Platelet	1.00 (0.99, 1.01)	0.653		
Neutrophil/lymphocyte ratio	1.21 (0.86, 1.68)	0.271		

*BCVA* best corrected visual acuity, *DR* diabetic retinopathy, *OR* odds ratio, *CI* confidence interval, *BMI* body mass index

*P < 0.05; **P < 0.01

**Table 3:** A model to predict DR-worsening

Parameters	Score Range	Estimates^a^
*Intercept*		1.15
Age	16–75	− 0.05
Baseline BCVA (logMAR)	− 0.08 to 1.00	2.08
*Diabetic nephrop*athy		
Yes	1	2.09
No	0	
*Hyperlipidemia*		
Yes	1	0.97
No	0	

*BCVA* best corrected visual acuity, *DR* diabetic retinopathy

^a^ Indicated the estimates were obtained from the multivariate regression logistic analysis of the four significant factors

A calculator was used to facilitate the utility of the model in clinical practice (Additional file 2). The calculation formula was developed as follows:

$$P (Y=1)=e^{(1.15-0.05x1+2.08x2+2.09x3+0.97x4)}/(1+e^{(1.15-0.05x1+2.08x2+2.09x3+0.97x4)} ).$$

In the model, $x$1 indicated age, $x$2 indicated baseline BCVA (logMAR), $x$3 indicated diabetic nephropathy, and $x$4 indicated hyperlipidemia. $P (Y=1)$ indicated the probability of DR-worsening as predicted by the model. An example was shown as follows: If a 52-year-old patient ($x$1 = 52) with a baseline BCVA (logMAR) of 1.00 ($x$2 = 1.00) and hyperlipidemia ($x$4 = 1) and without diabetic nephropathy ($x$3 = 0), then the predicted probability of DR-worsening was $P (Y=1)=e^(1.15-0.05x1+2.08x2+2.09x3+0.97x4) /(1+e^(1.15-0.05x1+2.08x2+2.09x3+0.97x4) )$= 83.20%.

### Model validation

**Fig. 2:**
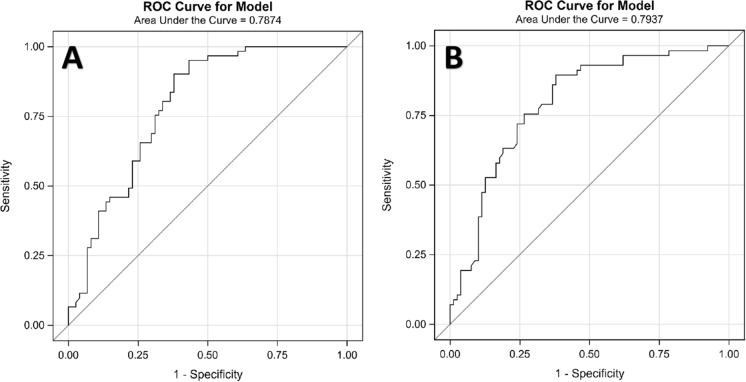
The area under the receiver operating characteristic (AUROC) curve for the model: **A** the training cohort; **B** the validation cohort

**Fig. 3:**
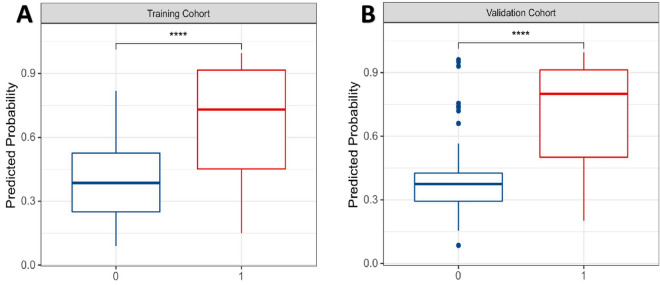
Boxplots of predicted probabilities in the two models: **A** the model with training cohort (Slope = 0.28); **B** the model with validation cohort (Slope = 0.29). The discrimination slope was defined as the difference between the mean predicted probability with DR-worsening (1) and non-DR-worsening (0)

The AUROC of the prediction model was 0.79 in the training cohort (Fig. 2A) and 0.79 in the validation cohort (Fig. 2B), the discrimination slope was 0.28 (95% CI 0.20–0.35) in the training cohort and 0.29 (95% CI 0.21–0.37) in the validation cohort (Fig. 3), illustrating good discriminative ability of the prediction model. Compared with 71.90% in the training cohort, the CCR was 71.30% in the validation cohort. Other metrics including sensitivity and specificity are shown in Table 4.

**Table 4:** Discrimination performances of the model

Cohort	AUROC	Slope	95% CI	*P* value	CCR	Sensitivity	Specificity
Training cohort	0.79	0.28	0.20–0.35	< 0.001	71.90%	52.70%	95.10%
Validation cohort	0.79	0.29	0.21–0.37	< 0.001	71.30%	59.50%	87.70%

*AUROC* area under the receiver operating characteristic curve, *CI* confidential interval, *CCR* correct classification rate

**Fig. 4:**
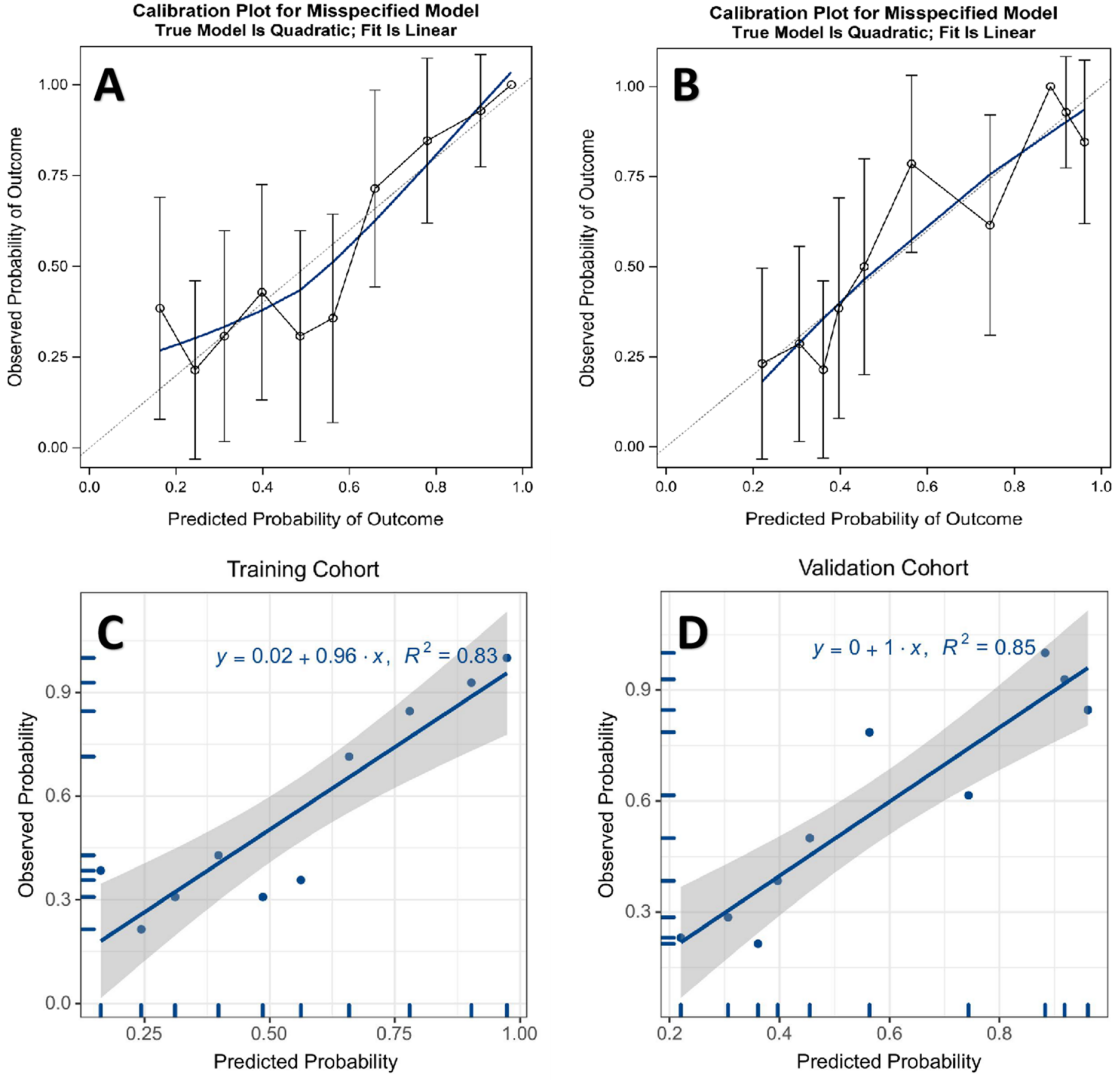
Plotting deciles of the predicted probability of DR-worsening against the observed probability for the model: **A**, **C** the training cohort; **B**, **D** the validation cohort. The x-axis is the predicted risk and the y-axis is the actual risk. The blue solid lines indicate the performance of the model, and a closer fit to the diagonal dotted lines indicates a better prediction

When considering the calibration ability of the model, the calibration slopes in the training and validation cohort were 0.96 (95% CI 0.60–1.31) (Fig. 4A, C) and 1.00 (95% CI 0.66–1.34) (Fig. 4B, D) respectively, the X-intercept and Y-intercept were both very close to 0, indicating that model had good calibration ability. Moreover, the *P* values for Hosmer–Lemeshow goodness-of-fit tests were 0.34 and 0.84 in the training and validation cohort respectively (Table 5). A *P* value of more than 0.05 in the goodness-of-fit test indicates that the consistency between the predicted and observed probability is good. Conversely, a *P* value of less than 0.05 indicates poor consistency. In the present study, *P* values were both above 0.05 in the training and validation cohorts, representing favorable consistency between predicted and observed probability of DR-worsening.

**Table 5:** Calibration performances of the model

Cohort	Slope	95% CI	X-intercept	95% CI	Y-intercept	95% CI	R squared	Goodness-of-fit test
Training cohort	0.96	0.60 to 1.31	− 0.03	− 0.39 to 0.15	0.02	− 0.19 to 0.24	0.83	0.34
Validation cohort	1.00	0.66 to 1.34	0.00	− 0.31 to 0.17	0.00	− 0.22 to 0.21	0.85	0.84

*CI* confident interval, *X* x-axis, *Y* y-axis

### Risk stratification

According to the best cut-off value of the predicted probability, which was 47.11% and 62.15% in the training and validation cohort respectively, patients were divided into the low-risk group (0–55.00%) and high-risk group (above 55.00%) based on their mean value (55.00%) (Table 6). The predicted probability in the two groups was 35.31% and 82.29%, respectively. The corresponding actual probability was 34.90% (52/149) and 82.79% (101/122), respectively (*P* < 0.001). In two groups, the observed actual probabilities were similar to the predicted probabilities of DR-worsening, indicating that the classification was reproducible.

**Table 6:** Classification of low-risk and high-risk groups

Group	Patients (n = 271)	Predicted probability^a^	Actual probability^a^	*P*^b^ (chi-square)
Low-risk (0–55%)	149	35.31%	52/149 (34.90%)	< 0.001
High-risk (above 55%)	122	82.29%	101/122 (82.79%)	

^a^ The rate of DR-worsening

^b^ An actual probability of DR-worsening between the two risk groups

## Discussion

This study investigated predictors of DR-worsening after PRP. After adjusting for various confounders, younger age, lower baseline BCVA (or higher logMAR BCVA), diabetic nephropathy, and hyperlipidemia were found to be independent predictors of a higher probability of DR-worsening after PRP. These four risk factors were then incorporated and developed into a new model to predict the risk of DR-worsening following PRP treatment within five years, which is convenient for clinicians and healthcare strategy makers to use. In addition, patients can be divided into a low-risk and high-risk group based on the probability of DR-worsening predicted by the model, which helps identify patients at a high risk of developing DR-worsening and prevent further loss of visual function.

Age at the onset of diabetes has been proved to be one of the key factors in the development and progression of PDR [24]. Studies have shown that younger patients with PDR had a higher risk of visual loss than older patients, and the onset age of type 2 diabetes under 45 years old was an independent risk factor for the development and progression of PDR [25, 26]. Previous studies have shown that more severe retinal proliferation, greater surgical difficulty, and lower anatomical reduction success rate due to rapid progression of retinal neovascularization could be found in younger PDR patients undergoing vitrectomy [27, 28]. The present study also reached a similar conclusion that younger age was an independent risk factor for DR-worsening after PRP. In addition, younger patients have higher prognostic requirements and economic burden associated with visual loss compared to the elderly. Therefore, age may be an important but often underappreciated prognostic factor of DR in clinical practice.

This study showed that DR progressed significantly after PRP treatment when the baseline BCVA was low. Increased visual loss is associated with increased DR severity [24], and thus once DR progressed, active treatment such as PRP or intravitreal anti-VEGF injection was one of the best ways to reduce DR-related blindness [29]. However, in the case of severe retinal ischemia, diffusion of oxygen needed by macular may remain insufficient and even lead to macular edema in spite of PRP, causing lower vision [15, 30]. This finding is also in line with the study that lower vision is associated with the larger avascular zone area of foveal in DR patients [31]. Therefore, prevention of DR-worsening may be an important strategy to reduce DR-related vision loss, even blindness.

In our study, the association of diabetic nephropathy with DR-worsening after PRP was observed to be statistically significant (*P* < 0.05 in both univariate and multivariate logistic regression analysis). In addition, the laboratory parameters related to renal function including creatinine, urea, and serum cystatin C had a statistically significant association with DR-worsening in the univariate logistic analysis, suggesting that with increasing severity of renal function there will be more likelihood of the DR-worsening. Furthermore, compared with the non-DR-worsening group, the patients in the DR-worsening group had worse kidney function and a greater frequency of diabetic nephropathy. Current studies have confirmed that diabetic nephropathy is closely related to DR, especially PDR or severe NPDR in diabetic patients [32–34]. Similarly, diabetic nephropathy was found to be an independent risk factor of DR-worsening after PRP. The pathophysiology of both DR and diabetic nephropathy is similar. The development of DR and diabetic nephropathy influences and promotes each other, which supports the view that the two diseases share a common etiological basis, and emphasizes that the treatment and care of DR should be combined with a multidisciplinary integrated treatment management model [35].

Our study also found that hyperlipidemia was the risk factor for the presence of DR-worsening after PRP treatment. In recent years, hyperlipidemia has been considered one of the strongest risk factors for the occurrence and development of DR [36, 37]. As reported in some studies, lipid-lowing therapy reduced the progression of DR and the need for laser treatment [38, 39], and total cholesterol and low-density lipoprotein were risk factors for the occurrence of any DR [23]. In addition, poor control of serum triglycerides was associated with progression of PDR [40], indicating that intensive lipid control might be associated with better clinical prognosis of DR after PRP treatment.

There were some prediction models about progression of DR [41, 42] or complications of diabetes, such as diabetic nephropathy[43] and diabetic foot [44]. To the author’s knowledge, no prediction model for DR patients with PRP treatment has been published, and studies on risk factors of the prognosis of PRP are also very scarce. Our model has four risk factors that are easy to obtain in medical records and further explores the interaction between these risk factors and DR-worsening, which have rarely been reported in previous studies and will provide a reference for future studies. Furthermore, the model can provide patients with an immediate and reliable assessment of DR-worsening within five years after PRP treatment. This estimation could guide clinicians to identify ones at a high risk of DR-worsening at an early stage and prescribe additional treatment, such as more frequent follow-up, supplemental laser photocoagulation therapy, or intravitreal anti-VEGF injection.

Nonetheless, the present study still had several limitations. Firstly, some studies have suggested that poor blood glucose control, long diabetes duration, hypertension, anemia, and other variables were also independent risk factors for DR-worsening [18, 45, 46]. However, this study did not produce similar results, possibly because patients with stable DR tended to lack regular review and even lose follow-up, which resulted in fewer patients in the non-DR-worsening group than in the DR-worsening group, and this might have introduced bias. In addition, this study was a retrospective analysis without standard diagnostic tests on patients among different doctors, and so was the collection of patient’s comorbidities. Lastly, while this model is useful in internal validation, external validation is also necessary. Therefore, prospective and multicenter studies are warranted to confirm these findings.

## Conclusion

In this study, the four independent risk factors, younger age, lower baseline BCVA (or higher logMAR BCVA), diabetic nephropathy, and hyperlipidemia, were found to be related to a higher probability of DR-worsening after PRP. This study developed and internally validated a new model to predict the probability of DR-worsening after PRP treatment within five years. The model can be used as a rapid risk assessment system for clinical prediction of DR-worsening and identify individuals at a high risk of DR-worsening at an early stage and prescribe additional treatment.

## Supplementary Information

**Additional file 1.** **Table S1** Characteristic comparison between the training and validation group. **Table S2** COX regression analysis of total patients.

**Additional file 2.** A calculator to predict DR-worsening within five years after PRP.

## Data Availability

Because we did not acquire consent from the patients in this study to share individual data publicly, health data of individuals cannot be available online for the public. However, the datasets used and analyzed during the current study are available from the corresponding author on reasonable request.

## Abbreviations

AUROC: Area under the receiver operating characteristic curve
BCVA: Best corrected visual acuity
BMI: Body mass index
CCR: Correct classification rate
CI: Confidence interval
DR: Diabetic retinopathy
NPDR: Non-proliferative diabetic retinopathy
OR: Odds ratio
PDR: Proliferative diabetic retinopathy
PRP: Panretinal photocoagulation
VEGF: Vascular endothelial growth factor
