# Supplementary material for: A prediction model for worsening diabetic retinopathy after panretinal photocoagulation
Source: Diabetol Metab Syndr. 2022 Aug 26;14:124. doi: 10.1186/s13098-022-00892-z (PMC9419399; doi:10.1186/s13098-022-00892-z)
Supplement: Supplementary file 1 — Additional file 1. Table S1 Characteristic comparison between the training and validation group. Table S2 COX regression analysis of total patients. [file 13098_2022_892_MOESM1_ESM.doc]

| **Table S1** Characteristic comparison between the training and validation group. | | | |
| --- | --- | --- | --- |
| Variables | Cohort, No. (%) | | *P* value^a^ |
|  | Training (N=135) | Validation (N=136) |  |
| Age (years) | 50.25±12.22 | 51.12±10.20 | 0.640 |
| Male, n (%) | 55.56% (75/135) | 61.03% (83/136) | 0.361 |
| Follow-up time, months | 47.65±45.16 | 42.73±35.44 | 0.931 |
| Baseline BCVA (logMAR) | 0.30 (0.10,0.53) | 0.30 (0.13,0.52) | 0.580 |
| Diabetes duration, years | 11.67±6.58 | 12.80±8.06 | 0.381 |
| Type 2 Diabetes, n (%) | 95.56% (129/135) | 97.06% (132/136) | 0.738 |
| PDR, n (%) | 66.67% (90/135) | 58.09% (79/136) | 0.145 |
| Diabetic nephropathy, n (%) | 22.22% (30/135) | 30.88% (42/136) | 0.107 |
| Diabetic neuropathy, n (%) | 8.15% (11/135) | 8.09% (11/136) | 0.986 |
| Coronary heart disease, n (%) | 9.63% (13/135) | 8.09% (11/136) | 0.655 |
| Prior Stroke, n (%) | 2.96% (4/135) | 7.35% (10/136) | 0.103 |
| Hyperlipidemia, n (%) | 30.37% (41/135) | 25.00% (34/136) | 0.323 |
| Hypertension, n (%) | 71.11% (96/135) | 76.47% (104/136) | 0.512 |
| BMI, kg/m2 | 25.56±3.71 | 25.49±3.13 | 0.739 |
| Homocysteine, umol/L | 13.31±7.19 | 13.36±8.60 | 0.749 |
| Fasting blood glucose, mmol/L | 7.03±2.66 | 6.89±2.90 | 0.372 |
| Urea, umol/L | 6.56±3.32 | 6.76±4.34 | 0.757 |
| Creatinine, mmol/L | 69.90 (56.70, 89.30) | 72.10 (56.78, 98.13) | 0.678 |
| Uric acid, umol/L | 328.36±87.32 | 327.61±93.36 | 0.946 |
| Total cholesterol, mmol/L | 4.39±1.18 | 4.33±1.18 | 0.430 |
| Triglyceride, mmol/L | 1.27 (0.93, 1.97) | 1.16 (0.85, 1.57) | 0.193 |
| High-density lipoprotein, mmol/L | 1.13±0.36 | 1.13±0.32 | 0.799 |
| Low-density lipoprotein, mmol/L | 2.73±0.95 | 2.78±1.00 | 0.958 |
| Serum superoxide dismutase, U/ML | 145.04±23.78 | 147.42±29.92 | 0.227 |
| Glycosylated serum protein, umol/L | 235.26±74.27 | 222.60±75.54 | 0.153 |
| Serum cystatin C, mg/L | 1.03 (0.89, 1.23) | 1.06 (0.86, 1.27) | 0.608 |
| Hemoglobin, g/L | 128.96±18.69 | 130.20±19.18 | 0.592 |
| Hematocrit | 0.38±0.05 | 0.38±0.05 | 0.235 |
| Platelet, 10^9^/L | 212.01±57.97 | 218.34±64.52 | 0.589 |
| Neutrophil/lymphocyte ratio | 1.91 (1.51, 2.53) | 1.93 (1.42, 2.62) | 0.893 |
| ^a^ For comparison between the training cohort and validation cohort. BCVA best corrected visual acuity; BMI body mass index; PDR proliferative diabetic retinopathy. | | | |

| **Table S2** COX regression analysis of total patients | | |
| --- | --- | --- |
| Variable | HR (95%IC) | *P* |
| Age | 0.98 (0.96, 1.00) | 0.029* |
| Sex | 0.92 (0.58, 1.44) | 0.709 |
| Baseline BCVA (logMAR) | 4.07 (2.47, 6.70) | ＜ 0.001** |
| Diabetes duration | 0.99 (0.97, 1.02) | 0.675 |
| Diabetes type | 1.39 (0.56, 3.46) | 0.483 |
| Stage of DR | 0.97 (0.67, 1.42) | 0.885 |
| Diabetic nephropathy | 2.35 (1.37, 4.03) | 0.002** |
| Diabetic neuropathy | 1.29 (0.73, 2.30) | 0.379 |
| Coronary heart disease | 0.78 (0.43, 1.43) | 0.426 |
| Prior Stroke | 0.57 (0.26, 1.26) | 0.163 |
| Hyperlipidemia | 1.67 (1.02, 2.72) | 0.040* |
| Hypertension | 0.96 (0.81, 1.13) | 0.620 |
| BMI | 1.00 (0.95, 1.06) | 0.930 |
| Homocysteine | 0.99 (0.97, 1.02) | 0.548 |
| Fasting blood glucose | 0.95 (0.88, 1.03) | 0.227 |
| Urea | 1.06 (0.98, 1.15) | 0.153 |
| Creatinine | 1.00 (1.00, 1.00) | 0.270 |
| Uric acid | 1.00 (1.00, 1.00) | 0.705 |
| Total cholesterol | 1.00 (0.56, 1.81) | 0.990 |
| Triglyceride | 1.13 (0.80, 1.59) | 0.489 |
| High-density lipoprotein | 1.73 (0.74, 4.07) | 0.208 |
| Low-density lipoprotein | 0.96 (0.53, 1.76) | 0.900 |
| Serum superoxide dismutase | 1.00 (0.99, 1.01) | 0.647 |
| Glycosylated serum protein | 1.00 (1.00, 1.00) | 0.295 |
| Serum cystatin C | 1.18 (1.00, 1.40) | 0.056 |
| Hemoglobin | 1.03 (0.98, 1.07) | 0.263 |
| Hematocrit | 0.00 (0.00, 810.80) | 0.269 |
| Platelet | 1.00 (1.00, 1.00) | 0.886 |
| Neutrophil/lymphocyte ratio | 1.04 (0.91, 1.20) | 0.571 |
| BCVA best corrected visual acuity; BMI body mass index; DR diabetic retinopathy; HR hazard ratio; CI confidence interval  **P* < 0.05, ***P* < 0.01. | | |
